# MRI pelvis screening to guide treatment of pelvic pathology

**DOI:** 10.1186/2050-5736-3-S1-P87

**Published:** 2015-06-30

**Authors:** Kelli Bryant, Suzanne LeBlang

**Affiliations:** 1 University MRI, Boca Raton, Florida, United States

## Background/introduction

The purpose of this retrospective study is to examine the incidence and imaging appearance of pelvic malignancies in a symptomatic patient population being screened for MRgFUS treatment.

## Methods

373 women with symptomatic uterine fibroids were screened with MRI exams with and without contrast utilizing T2 coronal and axial, T2 fat suppressed sagittal, T1 axial precontrast images, and post contrast fat saturated images in 3 planes. Uterine masses were classified by their intensity on T2 weighted images relative to normal myometrium (hypointense, isointense, or hyperintense as well as tissue homogeneity or heterogeneity). The enhancement pattern was also categorized as homogenous or inhomogeneous. Any extra-uterine masses were also noted as well as any other incidental pelvic findings.

## Results and conclusions

Results: Of the 373 patients (ages 26-61) that underwent pelvic screening for MRgFUS treatment, 19 presented with findings suspicious for cancer and after further evaluation, 7 of these patients (1.9 %) were confirmed to have a pelvic malignancy. 5 patients had a uterine sarcoma, 1 had endometrial carcinoma and 1 had ovarian carcinoma. Three out of the five sarcomas appeared markedly heterogeneous on T2 weighted images with ill-defined dark areas and bright fluid components and demonstrated heterogeneous, poorly defined areas of enhancement throughout. The remaining 2 sarcomas appeared more fluid in nature with eccentric, enhancing soft tissue components and multiple septations.

The endometrial carcinoma appeared as an intra-cavitary mass with ill-defined margins with a blood clot in the endometrium. This finding is highly unusual, as a large endometrial clot is not typically seen in cases where irregular bleeding was noted for fibroids. The ovarian carcinoma appeared as an extra-uterine mass with metastatic spread to the pelvic lymph nodes. Malignancy was considered in the remaining 12 patients due to the MRI imaging characteristics listed in Table 1 below. Three of these patients were excluded from MRgFUS treatment, but in retrospect had non-suspicious findings. Contact information was not available to obtain a definitive diagnosis for the other 9 patients. Conclusions: 1.9% of patients with suspected fibroid disease were found to harbor a pelvic malignancy following MRI screening exams for MRgFUS. These findings suggest that before any treatment for pelvic pathology, MRI screening should be used to guide physicians to the appropriate treatment modality (surgical *vs*. minimally invasive procedures). It is equally important to avoid delayed diagnosis and unintended treatment of malignant diseases with non-excisional approaches at this time. MRI pelvis exams may also help direct patients with more typical benign uterine fibroids to both non-invasive treatments (MRgFUS or uterine artery embolization (UAE)) or surgery (laparoscopy or open surgical procedures). The more liberal use of MRI pelvis exams to screen and characterize pelvic pathology in symptomatic women can help avoid recent deleterious outcomes with morcellation procedures of unsuspected malignancies. With increasing familiarity with the MR appearance of uterine pathology and attentive evaluation of screening studies, early diagnosis of pelvic malignancy may be achieved.

**Table 1:** Patients excluded from MRgFUS treatment due to the possibility of underlying malignancy.

Reason for Suspected Malignancy	Number of Patients
**Heterogeneous enhancement with ill-defined areas of hypo-enhancement**	3

**Overcall**	3

**Intra-cavitary mass w/blood products and heterogeneous enhancement**	2

**Intra-cavitary masswith homogenous enhancement**	2

**Ill-defined borders**	1

**Heterogeneous enhancement with well-defined hypo-enhancement**	1

**Figure 1:**
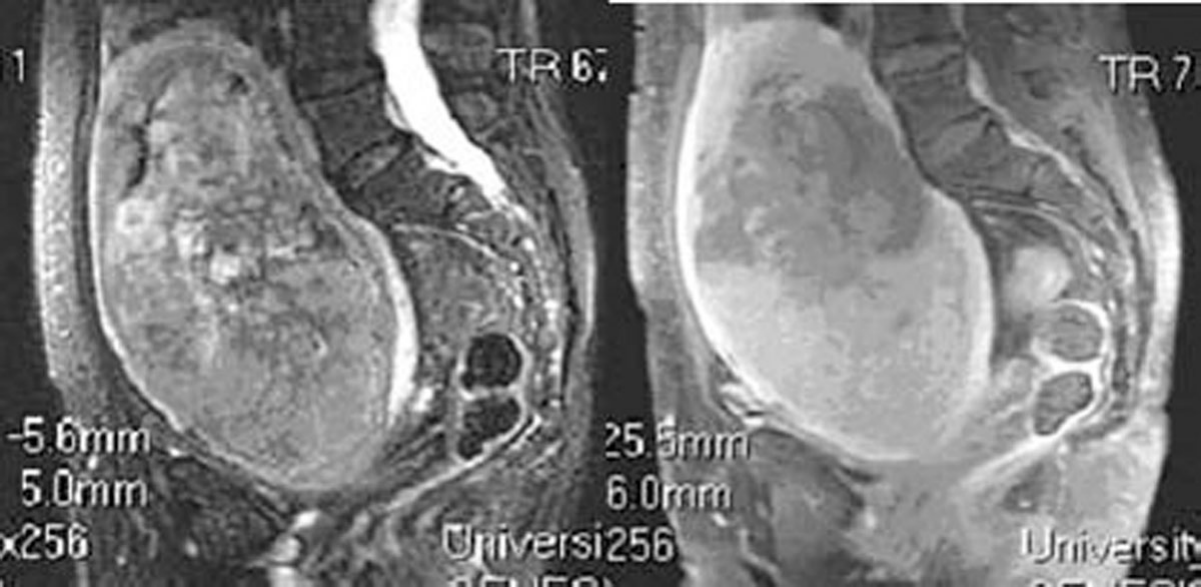
Sagittal T2-fat suppressed image depicting a markedly heterogeneous uterine sarcoma.

